# Deer activity levels and patterns vary along gradients of food availability and anthropogenic development

**DOI:** 10.1038/s41598-024-60079-6

**Published:** 2024-05-03

**Authors:** Zackary J. Delisle, Richard D. Sample, Joe N. Caudell, Robert K. Swihart

**Affiliations:** 1 Indiana Department of Natural Resources, Bloomington, IN 47401 USA; 2 Department of Forestry and Natural Resources, Purdue University, West Lafayette, IN 47907 USA; 3 Brownstown Ranger District, Hoosier National Forest, Bedford, IN 47421 USA; 4 Present Address: Arctic Inventory and Monitoring Network, National Park Service, AK 99709 Fairbanks, USA

**Keywords:** Behavioural ecology, Macroecology

## Abstract

Animal activity reflects behavioral decisions that depend upon environmental context. Prior studies typically estimated activity distributions within few areas, which has limited quantitative assessment of activity changes across environmental gradients. We examined relationships between two response variables, activity level (fraction of each day spent active) and pattern (distribution of activity across a diel cycle) of white-tailed deer (*Odocoileus virginianus*), with four predictors—deer density, anthropogenic development, and food availability from woody twigs and agriculture. We estimated activity levels and patterns with cameras in 48 different 10.36-km^2^ landscapes across three larger regions. Activity levels increased with greater building density, likely due to heightened anthropogenic disturbance, but did not vary with food availability. In contrast, activity patterns responded to an interaction between twigs and agriculture, consistent with a functional response in habitat use. When agricultural land was limited, greater woody twig density was associated with reduced activity during night and evening. When agricultural land was plentiful, greater woody twig density was associated with more pronounced activity during night and evening. The region with the highest activity level also experienced the most deer-vehicle collisions. We highlight how studies of spatial variation in activity expand ecological insights on context-dependent constraints that affect wildlife behavior.

## Introduction

Individual animals must decide when and for how long to remain active each day. Such decisions can depend on a complex array of potentially antagonistic forces including duration of daylight^1^, food availability^2^, reproduction or rearing of young^3^, and temporal pulses in predation risk or fear^4^. Theory predicts that animal decisions on activity strive to balance these factors to maximize energetic efficiency by remaining active as little as possible while simultaneously obtaining necessary nourishment and minimizing predation risk^5^.

Accurate estimation of activity level (i.e., how much of a day spent active) and pattern (i.e., when activity occurs) of animal populations requires sampling across the landscape that animals use throughout the day. If some used landcover types are not sampled, activity metrics may be biased and misleading. Camera traps have been used commonly to estimate activity metrics because they are able to sample continuously within a wide variety of landcover types^6^.

Unfortunately, the intensity of sampling needed to estimate several replicate activity distributions poses logistical difficulties in terms of purchasing and deploying camera traps, and classifying animals within captured images. Quantitative study of how activity changes across space is thus challenging and rare. Most comparisons of animal activity metrics have been restricted to qualitative comparisons of visual activity graphs and statistical tests between two activity levels or patterns at once^6^. More detailed examinations of how activity quantitatively changes across multiple explanatory variables, landscapes, and regions would improve our ecological understanding of behavioral ecology.

The white-tailed deer (*Odocoileus virginianus*; henceforth, deer) is a common New World ungulate that inhabits diverse landscapes^7^. Deer behavior changes along environmental gradients including, but not limited to, human development^8^, food availability or quality^9^, and density of conspecifics^10^. Like other previous activity research, field studies of deer activity have been predominantly descriptive in nature or only included statistical comparisons of two activity distributions^11,12^. Because deer can exhibit behavioral fluidity in response to environmental context, additional plasticity in activity might be expected. We sought quantitative understanding of how deer activity levels and patterns change across gradients of various environmental characteristics during the winter in the midwestern USA (henceforth, Midwest).

Frequent disturbances may generally cause higher activity levels due to avoidance of perceived danger. Disturbances from human activities can influence numerous deer behaviors^13–15^. Human development in the form of roads and buildings may specifically affect deer activity because development is consistently associated with mortality from deer-vehicle collisions^16^. Therefore, deer may change their activity along gradients of human activity or development.

Deer in the Midwest rely on several food resources during winter including low-quality woody twigs in forested sites and higher-quality crop residue from agricultural fields after harvest. But deer also must balance food acquisition with predation risk^17^. According to foraging theory, deer should bias feeding activity in favor of safer areas and either exhibit greater per capita vigilance or form larger groups in riskier areas^18^. Fear of predation in deer may be associated with time of day and vegetative concealment (i.e., how much vegetation is obstructing direct line of sight to deer^19^). Within home ranges, disproportionate changes in habitat use with availability (i.e., functional responses in habitat use^20^) may result from attempts by individuals to balance decisions about activity with spatially varying risks and rewards^21^. In Midwest landscapes, deer use of crop residue in open agricultural fields and woody twigs in forest patches may reflect attempts to balance tradeoffs of forage quality, quantity, or perceived predation risk. If so, interactions between the amount of agriculture and woody twigs may be important predictors of deer activity.

Density dependence in ungulates is of interest ecologically and because of its implications for management of game species^10,22^. Theory suggests that larger densities of ungulates can decrease the amount of available per capita food and thereby increase intraspecific competition for nutrients^23^. Density-dependent responses in activity of wild boar (*Sus scrofa*), red deer (*Cervus elaphus*), and roe deer (*Capreolus capreolus*) have been documented^24^. Moreover, elk (*Cervus canadensis*) exhibited density-dependent habitat selection^25^. But no quantitative assessment has examined how deer activity shifts as a function of population density.

### Hypotheses and predictions

Our goals were to determine whether and to what extent deer winter activity levels and patterns vary spatially, and to discover if variables related to anthropogenic disturbances, food, and intraspecific density correspond to these activity changes. First, we hypothesized that deer would alter their activity levels in response to human development. We predicted that road length and density of buildings would exhibit positive relationships with deer activity levels.

Secondly, we hypothesized that deer activity levels would respond to intraspecific density. Greater density, by decreasing available per capita food, may increase the amount of time spent foraging^23^; accordingly, we predicted a positive relationship between activity levels and intraspecific density of deer.

Thirdly, we hypothesized that deer activity levels would respond to food availability. We predicted that deer activity levels would decrease as the density of woody twigs and the amount of agricultural land increased, because more available food should decrease the time required for foraging. Further, we predicted a significant interactive effect of woody twigs and agricultural land due to activity levels of deer requiring tradeoffs between resource acquisition and safety.

Fourthly, we hypothesized that deer activity patterns would respond to availability of different food types in a manner that incorporated time-dependent tradeoffs between forage acquisition and safety. Deer are crepuscular^26^, predominantly consuming agricultural products in open fields during nocturnal hours but consuming woody twigs during any time of day^27^. Moreover, deer may prefer diverse diets which can ensure proper nutrient intake and avoidance of overconsumption of any single mineral^28,29^. Therefore, in areas with small amounts of agricultural land, we predicted deer would alter their activity patterns to be more active at night in areas where woody twig density was low, because deer would rely more heavily on crops as a food source at night. In contrast, we predicted deer would be less active at night in areas where woody twig density was high, because deer would be able to consume adequate amounts of twigs during daytime in the relative safety of woodlands. However, in areas with plentiful amounts of agricultural land, we predicted deer would be more active during daytime in areas where woody twig density was low, because deer would need to search for and consume scarce woody twigs throughout daytime. In areas with plentiful agricultural land, we also predicted deer would be more nocturnal in areas where woody twig density was high, because woody twigs would be readily available for consumption during daytime and thus deer could concentrate their time spent active during nocturnal hours when crop consumption could more safely occur.

## Results

We sampled 1,018 unique locations with camera traps (total trap nights = 13,816). Cameras did not detect deer at 187 locations. The average number of sampling locations and deer detections used for activity estimation was 21 and 536, respectively, per replicate 10.36-km^2^ landscape. We detected > 100 deer detections in 42 different landscapes.

### Regional analysis

**Figure 1:**
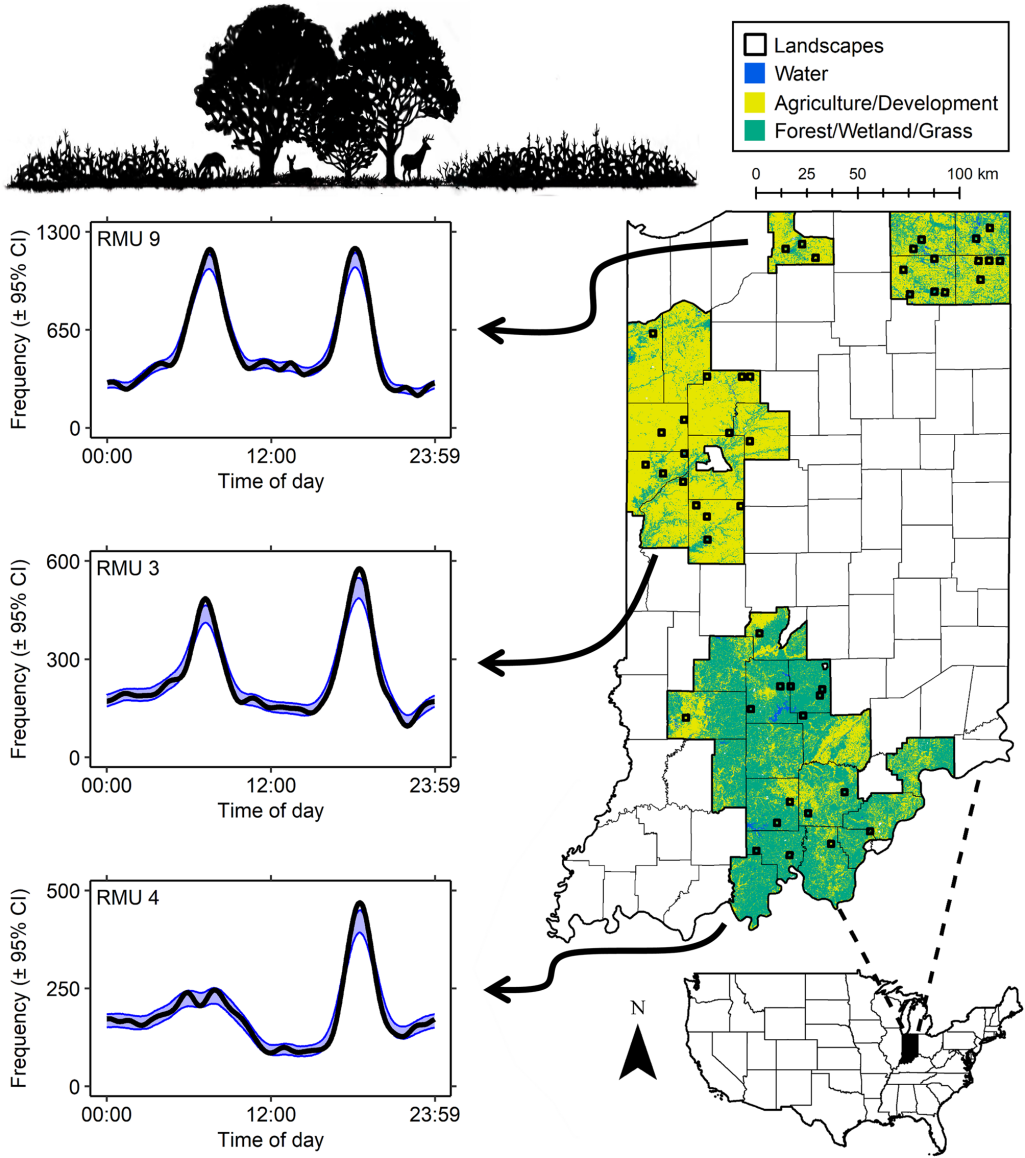
Landcover types and sampled landscapes within Regional Management Units (RMU) 3 (west central), 4 (southern), and 9 (northeastern, two separate areas) within Indiana, USA. Within each RMU, we estimated the activity distributions of white-tailed deer using camera traps deployed within landscapes during the winters from 2019 to 2021.

Activity distributions in RMUs 3 and 9 both exhibited bimodal peaks characteristic of crepuscular activity, whereas activity in RMU 4 peaked predominantly during the evening and to a much lesser extent in the morning (Fig. 1). Both activity levels and patterns varied significantly across regions. We estimated activity levels of 0.41 (standard error [SE] = 0.01), 0.39 (SE = 0.02), and 0.44 (SE = 0.01) in RMU 3, 4, and 9, respectively. Activity level in RMU 3 was similar to RMUs 4 (observed difference = 0.02, SE = 0.02, W = 1.27, P = 0.26) and 9 (observed difference = 0.03, SE = 0.02, W = 2.60, P = 0.21). However, differences arose in activity levels between RMUs 4 and 9 (observed difference = 0.05, SE = 0.02, W = 8.60, P = 0.010).

**Figure 2:**
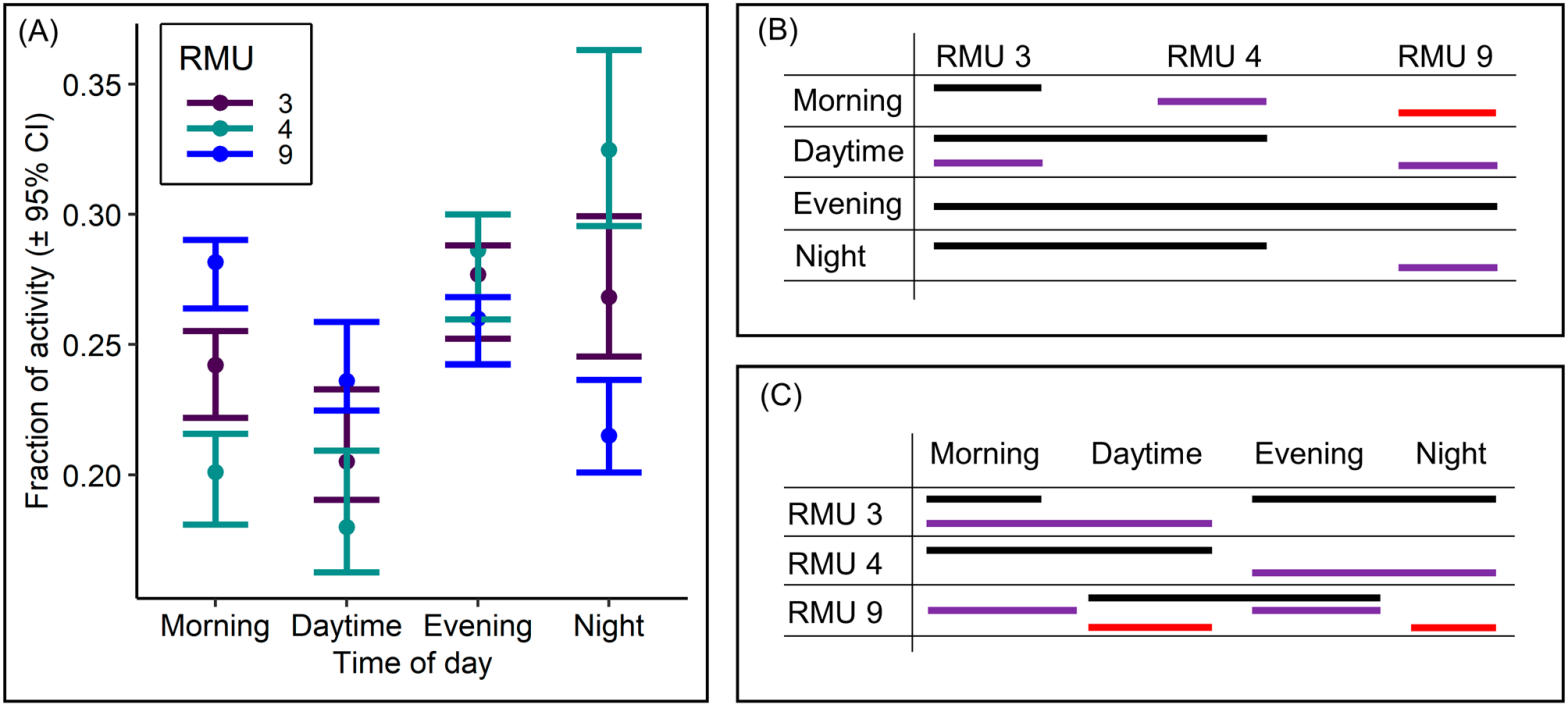
The fraction of deer activity levels (± 95% confidence intervals) observed during morning, daytime, evening, and night within Regional Management Units (RMU) 3, 4, and 9 in Indiana, USA (**A**). Fractions of deer activity levels observed between RMUs and within times of day (**B**) or within RMUs and between times of day (**C**) are similar (P > 0.05) if sharing an identically colored line. We collected activity data using camera traps during the winters of 2019 to 2021.

Deer spent more time active during morning and less at night in RMU 9, but the reverse was true for RMU 4 (Fig. 2). Deer allotted their activity more evenly throughout each of the four time-of-day categories in RMU 3 when compared to the other regions. Deer devoted similar fractions of total activity to evening hours in all three regions.

### Replicate landscape analysis

#### Activity levels

**Figure 3:**
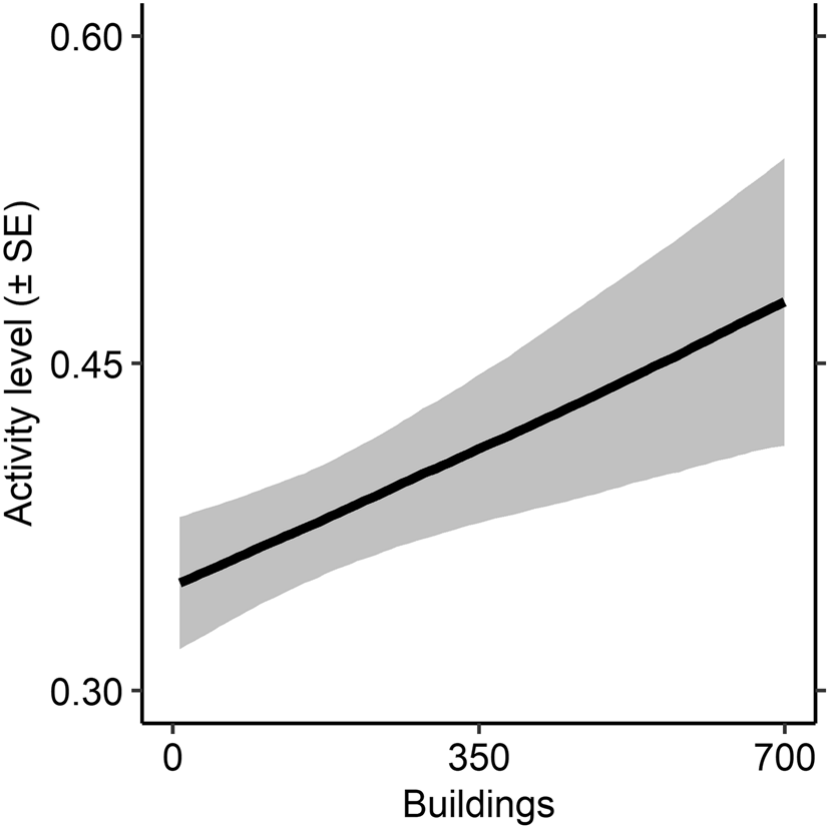
Effects plot from a hierarchical Bayesian beta regression model predicting white-tailed deer activity levels (± standard error) as a function of the number of buildings within 3.2 × 3.2-km landscapes. Activity data were collected using camera traps during the winters of 2019 to 2021 in Indiana, USA.

Models containing predictors associated with anthropogenic development, twig density, and intraspecific density had evidence ratios < 3; neither area of agriculture nor models with interactions received support (Table 1, Table S1). The model containing number of buildings had the most support; in it, the number of buildings exhibited a strong positive relationship with deer activity levels (Table 2, Fig. 3).

**Table 1:** Support for models regressing white-tailed deer activity levels (Response = Level) and the fraction of activity level exerted during different times of day (Response = Pattern).

Response	Hypothesis	Predictor	Weight	Evidence ratio
Level	Anthropogenic disturbance	Buildings	0.362	1.000
	Food	Roads	0.243	1.488
		Twigs	0.129	2.812
		Agriculture	0.108	3.340
		Twigs*agriculture	0.020	18.274
	Density dependence	Deer density	0.138	2.618
Pattern	Food	Twigs	0.054	17.222
		Agriculture	0.016	58.125
		Twigs*agriculture	0.930	1.000

Model weights were based on approximate leave-one-out cross validation, and evidence ratios (i.e., the weight of the top model divided by the weight of the model under consideration) were computed using model weights. Weights for different responses were computed separately. Both deer activity metrics were estimated from camera trap data collected in Indiana during the winters of 2019 to 2021. Hypothesis = the general a priori reasoning for expecting an effect on activity. Predictor = the covariates contained within the model being considered. Buildings = the number of buildings in the landscape (i.e., 3.2 × 3.2-km area in which camera traps were deployed). Roads = the length of roads within the landscape. Twigs = the density of non-avoided woody twigs in the landscape. Agriculture = the amount of land used for agriculture in the landscape. Deer density = the average density of deer in the landscape. “*” denotes an interactive and additive effect.

**Table 2:** Estimates and 89% lower (LCI) and upper (UCI) confidence intervals for regression coefficients from all beta regression models of white-tailed deer activity levels across various predictors.

Model form	Predictor	Estimate	LCI	UCI
~ Buildings	Buildings	0.124	0.022	0.227
~ Roads	Roads	0.093	− 0.018	0.195
~ Twigs	Twigs	− 0.034	− 0.138	0.075
~ Deer density	Deer density	0.049	− 0.063	0.154

Only shown are models exhibiting evidence ratios < 3. Model form denotes the predictors used to fit the model. Predictors are defined in Table 1. Activity data were collected using camera traps during the winters of 2019 to 2021 in Indiana, USA.

#### Activity patterns

**Figure 4:**
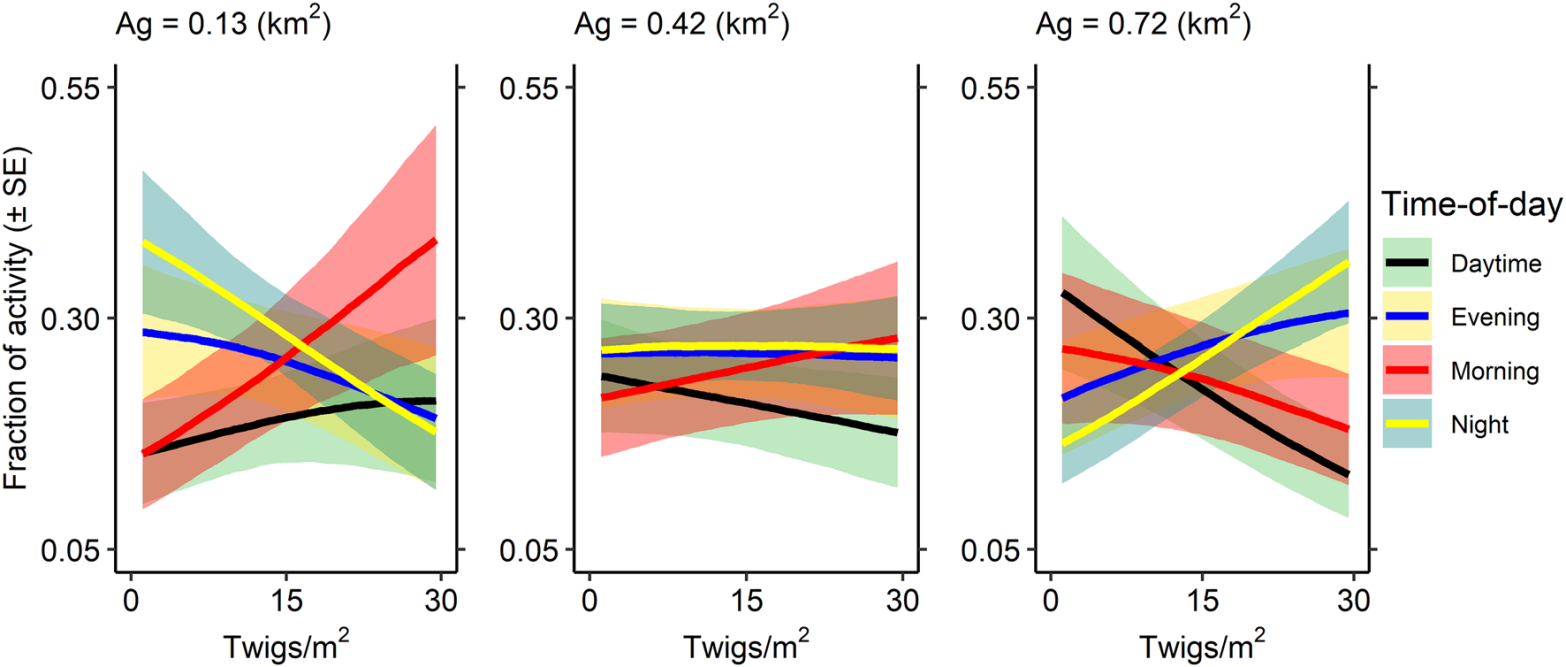
Effects plot from a hierarchical Bayesian Dirichlet model regressing the fraction of white-tailed deer activity levels exerted during night (baseline), morning, daytime, and evening (± standard error, SE) across an interaction between the amount of land used for agriculture (0.13, 0.42, and 0.72 km^2^ = − 1, mean, and 1 standard deviation, respectively) within the 3.2 × 3.2-km landscape and the density of woody twigs within the same landscape (twigs/m^2^). Activity data were collected using camera traps during the winters of 2019 to 2021 in Indiana, USA.

The only model with support for activity patterns contained a strong interaction between twig density and the amount of surrounding land devoted to agriculture (Tables 1, 3). Specifically, when the amount of agricultural land was small, increases in woody twig density were associated with reduced deer activity during night and evening periods, and greater relative activity during morning and (moderately) during daytime periods (Fig. 4). Conversely, when the amount of agricultural land was large, increases in woody twig density were associated with greater deer activity during night and evening periods, and reduced activity during morning and daytime periods (Fig. 4). We provide plots of posterior distributions, posterior predictive checks, and trace plots in Supplementary Figs. S1–S4.

**Table 3:** Estimates and 89% lower (LCI) and upper (UCI) confidence intervals for coefficients from a Dirichlet regression modeling the fraction of white-tailed deer activity levels exerted during night (baseline), morning, daytime, and evening across various predictors.

Time-of-day	Predictor	Estimate	LCI	UCI
Morning	Twigs	0.053	− 0.105	0.213
	Agriculture	0.067	− 0.146	0.279
	Twigs:agriculture	− 0.321	− 0.473	− 0.170
Daytime	Twigs	− 0.072	− 0.240	0.093
	Agriculture	0.187	− 0.020	0.400
	Twigs:agriculture	− 0.314	− 0.475	− 0.156
Evening	Twigs	− 0.008	− 0.159	0.147
	Agriculture	0.107	− 0.086	0.311
	Twigs:agriculture	− 0.090	− 0.239	0.063

Only one model is shown; no other models exhibited an evidence ratio < 3. Predictors are defined in Table 1. Activity data were collected using camera traps during the winters of 2019 to 2021 in Indiana, USA. Night was used as the baseline.

## Discussion

Activity patterns of deer responded to the interaction of natural and agricultural food sources, supporting our prediction. Other studies have demonstrated functional responses in habitat use by ungulates consistent with modulation of site-specific differences in risk and reward^30,31^. But our results are, to our knowledge, the first to document such modulation operating across landscapes in a manner that shifts the time of day during which activity occurs. In contrast to our prediction, we found no strong relationships between deer activity levels and these same sources of foods. Although we tested a large gradient of woody twig density and agricultural availability, food in our study areas may not have been limited enough to alter both activity levels and patterns simultaneously. Instead of changing activity levels, deer appeared to obtain sufficient amounts of both agriculturally and naturally sourced foods while adjusting their activity patterns based upon the availability of each food source.

Although previous research documented activity levels to be density dependent in other ungulates^24^, we found no strong relationship between deer density and deer activity levels. Limitation of food resources is a major contributor to intraspecific competition and ecological carrying capacity for deer^32^. But other research found minimal effects of deer use intensity (an index of population density) on the health of forest understories in the same areas and times we deployed camera traps^33^. Vast amounts of agricultural food are present in RMUs 3 and 9, and forests containing woody food are widespread in RMU 4. Therefore, plentiful food resources may have reduced or eliminated any potential intraspecific competition for food within our study system and thus negated our ability to detect any potential response in deer activity levels to food.

Deer activity may be more broadly related to other areas of interest in wildlife management. For instance, deer-vehicle collisions are a major concern of wildlife managers^34^. Population density has been a key focus of those attempting to reduce or model animal-vehicle collisions^35–37^, but animal activity may also play a role^38,39^. If animals either are active during a greater fraction of each day or shift their activity to coincide with periods of peak vehicular traffic volume, the chances of animals and vehicles colliding on the landscape likely will increase. In our study, we documented in RMU 9 the highest regional activity levels and a pattern characterized by a greater fraction of activity during the morning rush hours. Under such conditions, accidents involving collisions between motorists and deer might be expected. Indeed, deer-vehicle collisions occur at a rate 1.98 times higher in RMU 9 compared to RMU 3 and 4^40^. Therefore, quantitative examinations of the relationships between characteristics of activity distributions and deer-vehicle collisions may help future management planning to reduce collisions. If positive relationships are found, incentivizing humans to hunt deer in close proximity to roadways may reduce occurrence of deer-vehicle collisions by causing deer to shift to nocturnal activity patterns, reduce movement rates, or select areas further from roads^41,42^.

Metrics from estimated animal activity distributions are fundamental parameters used for research purposes in energetics^11^, population ecology^43^, movement ecology^44^, species interactions^11^, and urban ecology^45^. In population estimation, activity levels are often used to estimate the sampling availability (i.e., fraction of the 24-h day that animals are moving and thus able to be detected by camera traps) of the target species for camera trapping^38,46^. In so doing, most studies have used a single estimate of animal activity to correct for the sampling availability of the target population. However, our results suggest that models of variation in activity level across study regions may yield more accurate estimates of sampling availability. Improvements may be especially dramatic for spatially explicit models of density that depict how the size of animal populations change across space while also relying on estimates of sampling availability. We encourage future research in population ecology to consider how sampling availability changes across space.

Quantitative modelling of spatial variation in animal activity metrics requires sampling intensity sufficient to estimate activity distributions within many spatial replicates. Such modeling has the capacity to enhance our understanding of animal behavior, and to improve knowledge useful for wildlife management and conservation. The sampling effort required for modeling of animal activity poses both logistical and financial challenges. Fortunately, large-scale camera trapping operations are becoming more common in wildlife research and management^47–49^ and should enable closer inspection of environmental and human factors affecting animal activity. Moreover, opportunities to integrate other data sources (e.g., bio logging^50^) should improve our ability to detect shifts and test for effects of risk:reward tradeoffs on activity levels and patterns across environmental gradients.

## Methods

### Study sites

We deployed camera traps within Regional Management Units (RMU) 3, 4, and 9 in Indiana, USA^40^. We used the same sampling design and camera deployment strategy outlined in previous research within the same study areas^51^. We placed cameras in random 10.36-km^2^ sub-areas (henceforth, landscapes). We sampled 20 landscapes across all three RMUs in each year. We repetitively sampled two landscapes in each RMU during each year to examine annual differences unrelated to spatial variation. Thus 48 unique landscapes were sampled. A thorough description of each RMU is presented in previous research^52^.

### Data collection

We randomly deployed camera traps > 200 m apart within forests, wetlands, grasslands, and agricultural fields^53^. We affixed cameras at 1-m height to trees or metal poles that we hammered into the ground if trees were not present. We programmed cameras to capture a 3-photo burst when triggered, and specified a minimum delay between bursts of 1 s. We collected data from camera trap images during 2-week intervals from 12–25 February 2019, 9–22 March 2020, and 25 February–10 March 2021. After data collection, we classified photos containing deer and recorded the time of day of each encounter at each camera.

### Estimation of activity levels and patterns

Times of day when animals are active and inactive are associated with greater and lesser numbers of captures by camera traps, respectively^54^. The distribution of detection times can thus be used to estimate animal activity level. To do this, we first double-anchored detection times of deer based on the average sunrise and sunset times at camera trap locations on days we collected data using methods of previous research^1^. Double anchoring detection times accounts for the differing sunset and sunrise times across the days and locations that we sampled, which is critical because deer are crepuscular^26^. We then fit circular kernel probability density functions to the double-anchored detection times of deer using the methods of previous research^53^. We used the area under each circular kernel probability density function as the estimate of deer activity level, and used nonparametric bootstrapping to estimate uncertainty associated with activity levels^54^. We used the “activity” package in R to double anchor detection times, fit probability density functions, and estimate activity levels^55,56^.

We estimated activity patterns by first defining four different time-of-day categories: night (defined as > 2 h after sunset to > 2 h before sunrise), morning (defined as < 2 h before sunrise to < 2 h after sunrise), daytime (defined as > 2 h after sunrise to > 2 h before sunset), and evening (defined as < 2 h before sunset to < 2 h after sunset). We then used numerical integration to estimate what fraction of the daily deer activity level that occurred within each time-of-day category by

$${\int }_{{t}_{2}}^{{t}_{1}}f(x)dx$$

where ${t}_{1}$ and ${t}_{2}$ are the two temporal bounds of a given time-of-day category, and $f(x)$ is the circular kernel density used to model activity level.

### Predictors of activity

#### Deer density

We estimated spatially explicit deer density using the density surface model from a previous project in the same study areas and times this study was conducted^51^. These density estimates utilized camera trap distance sampling and landscape covariates within a generalized additive model to predict deer density inside 30 × 30-m cells across each of the RMUs we sampled^51^. To estimate deer density within each landscape, we averaged the density estimate of each cell within the landscape of interest. Density estimates within landscapes ranged from 0.78 to 37.43 deer/km^2^ (mean = 9.06, SE = 1.08).

#### Anthropogenic development

We calculated the total road length and number of buildings within landscapes using the Indiana primary and secondary roads state-based shapefile^57^ and the US building footprints shapefile from Microsoft (https://github.com/Microsoft/USBuildingFootprints), respectively.

#### Food availability

We calculated the total amount of agricultural land within landscapes using the National Land Cover Database 2019 landcover raster file^53^. We also estimated the density of non-avoided twigs as an index of natural food using the methods and data from previous research in the same study areas^58^. Specifically, we sampled five 1-m^2^ quadrats placed every 10 m along randomly placed and oriented 50-m transects. The number of transects sampled per forest patch was determined by ${A}_{i}/2{NT}_{i}<{NT}_{i}$, where ${NT}_{i}$ = the number of transects in forest patch $i$, and ${A}_{i}$ = the area (ha) of forest patch $i$. We counted all living woody twigs in 3-dimensional space 20–180 cm above the quadrat^59^, and estimated twig density (${D}_{i}$) by ${D}_{i}={t}_{i}/{n}_{i}$, where ${t}_{i}$ = the total number of twigs counted in forest patch $i$, and ${n}_{i}$ = the total number of quadrats surveyed within forest patch $i$. We used a Pearson’s chi-square test for count data^60^ to classify twig species as non-avoided, which we defined as significantly (consumed at a higher rate than expected; α ≤ 0.05) or neutrally (consumed at a similar rate than expected; α > 0.05) selected for consumption.

#### Natural predators and human hunting

Deer will often alter their decision making when they co-occur with predators or perceived predators^61,62^. Although coyotes (*Canis latrans*) predominantly prey solely on juvenile deer^63^, past research has found behavioral responses in deer when exposed to coyotes^64^. However, during times of the year when juvenile deer are extremely young, past research found only groups containing juveniles to respond to coyote presence by altering their activity pattern^11^. Groups solely containing older deer did not alter their activity patterns when subjected to the presence of coyotes^11^. Our study was conducted later in the year when juvenile deer are much larger and thus less susceptible to predation by coyotes. For these reasons, we did not consider coyotes as a predictor of deer activity patterns in this work.

Human hunting is a major source of deer mortality^16^ and can affect several deer behaviors^41,42^. However, outside of times of year or areas where humans hunt deer, deer often become accustomed to nonhunting humans (e.g., human walking or dog walking) and often do not perceive humans directly as risky^19,65^. Our sampling did not occur during seasons when humans could harvest deer in Indiana. For these reasons, we did not test the effects of indices of human hunters on deer activity levels or patterns.

### Regional analysis

We used a Wald χ^2^ statistic with 1 degree of freedom^54^ to test for differences between (1) activity levels within regions, (2) fraction of deer activity levels exerted within each time-of-day category between RMUs, and (3) fraction of deer activity levels exerted between each time-of-day category within RMUs. In each Wald test, $W=\frac{{{(AL}_{1}-{AL}_{2})}^{2}}{SE({{AL}_{1})}^{2}+SE({{AL}_{2})}^{2}}$, where ${AL}_{1}$ and ${AL}_{2}$ are the two activity levels being compared and $SE$ denotes the standard error. To minimize potential for Type 1 Error due to multiple pairwise comparisons, we implemented a Holm’s adjustment strategy^66^.

### Replicate landscape analysis

We modelled activity levels of deer within 10.36-km^2^ landscapes using a mixed effects beta regression. We used a beta regression because activity level is a fraction bounded between 0 and 1. We modelled the fraction of deer activity levels exerted during each of the four time-of-day categories using a mixed effects Dirichlet regression. We used a Dirichlet regression because the four fractions of deer activity levels exerted during each of the time-of-day categories are naturally compositional and thus sum to 1. We did not include activity levels or patterns from landscapes that had < 100 deer detections, as other research suggested that coefficients of variation exceed 0.10 with sample sizes of < 100 and thus are imprecise^54^.

We fit all models within a hierarchical Bayesian framework in R using the “brms” package^67^. Because landscapes were naturally nested within larger regions, we included a random intercept for region. For all continuous predictors, we subtracted the mean and divided by the standard deviation to aid convergence. We ran 3 Markov chains for a total of 2500 iterations per chain. We discarded the first 1000 iterations per chain and specified Student-T priors for all regression coefficients and random effects in the beta and Dirichlet models (degrees of freedom = 3, mean = 0, standard deviation = 2.5; default in “brms” package).

To examine support for each of the models, we used weights based on approximate leave-one-out cross validation^68^ and evidence ratios of model weights (i.e., the weight of the top model divided by the weight of the model under consideration). We considered models with evidence ratios < 3 to have statistical support for being the “best” model in the candidate set, and only further reported statistics on regression coefficients from these models. Because 89% confidence intervals are more stable when posterior samples are < 10,000, we considered strong predictors those with associated 89% credible intervals that did not overlap zero^69^.

### Supplementary Information

Supplementary Information 1.

Supplementary Information 2.

## Data Availability

The data used in this manuscript are included as Supplementary Information.
