# Supplementary material for: Deer activity levels and patterns vary along gradients of food availability and anthropogenic development
Source: Sci Rep. 2024 May 3;14:10223. doi: 10.1038/s41598-024-60079-6 (PMC11068751; doi:10.1038/s41598-024-60079-6)
Supplement: Supplementary file 1 — Supplementary Information 1. [file 41598_2024_60079_MOESM1_ESM.pdf]

SUPPLEMENTARY INFORMATION

Deer activity levels and patterns vary along gradients of food availability and anthropogenic development

Zackary J. Delisle[1, 2], Richard D. Sample[2, 3], Joe N. Caudell[1], and Robert K. Swihart[2]

[1] Indiana Department of Natural Resources, Bloomington, Indiana 47401, USA.
[2] Department of Forestry and Natural Resources, Purdue University, West Lafayette, Indiana 47907, USA.
[3] Brownstown Ranger District, Hoosier National Forest, Bedford, Indiana 47421 USA.

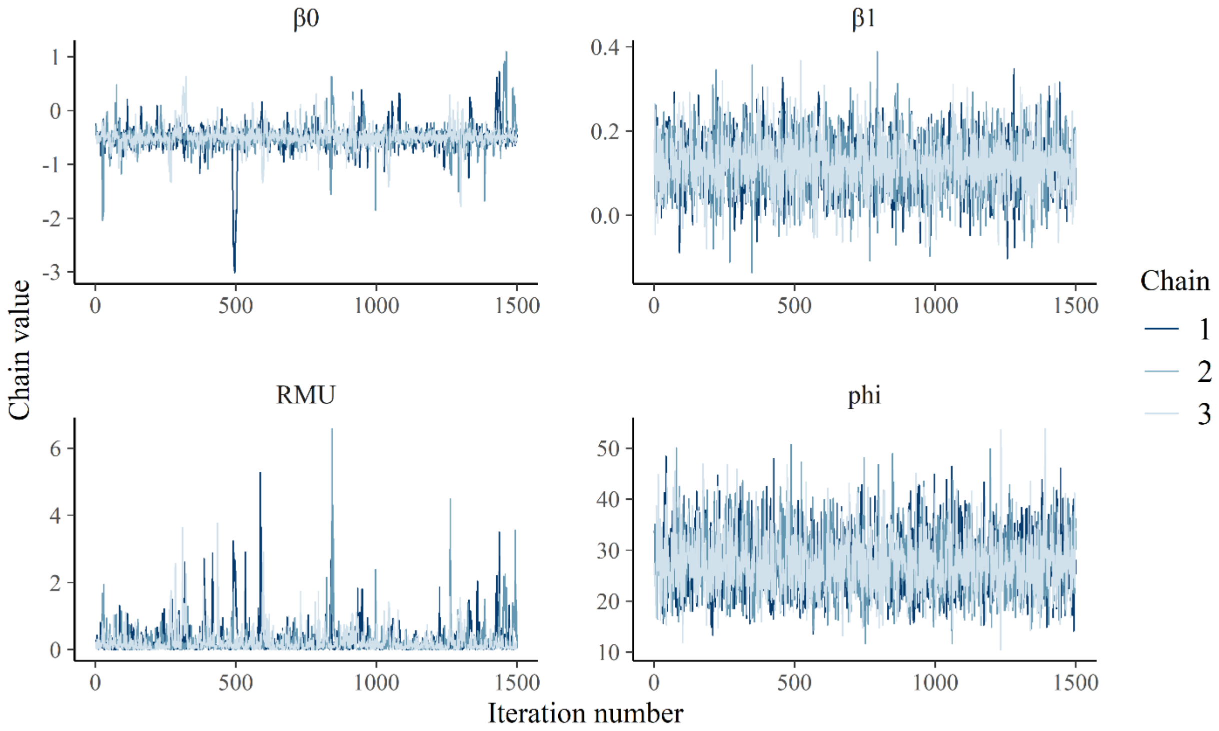

Supplementary Figure S1. Trace plots of parameters from a Bayesian beta regression model fitting activity levels of white-tailed deer within 3.2 x 3.2-km landscapes to the number of buildings within the same landscapes. β0 = intercept. β1 = number of buildings. RMU = random effect for Regional Management Unit.

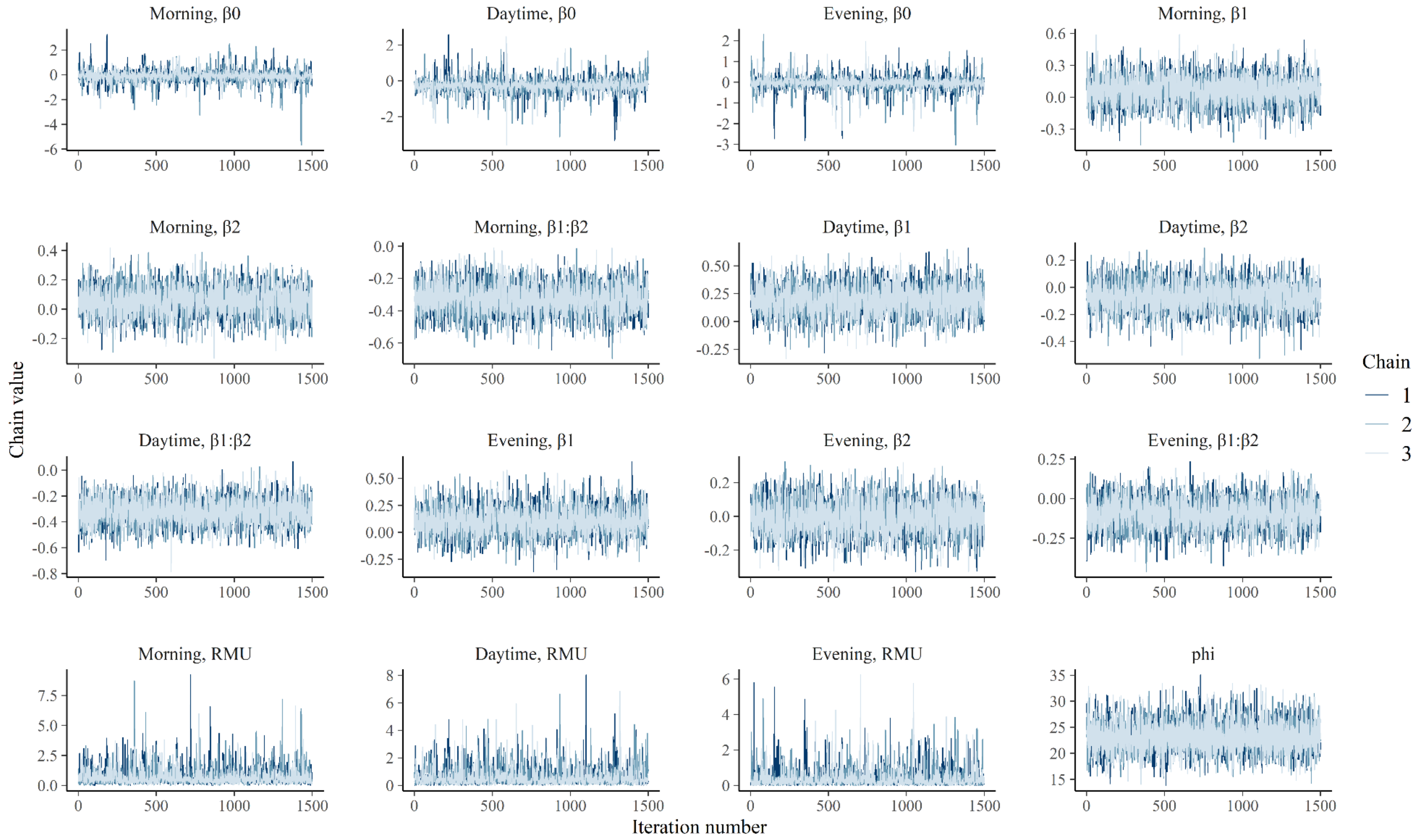

Supplementary Figure S2. Trace plots of parameters from a Bayesian Dirichlet regression model fitting activity levels of white-tailed deer exerted within morning, daytime, evening, and night within a 3.2 x 3.2-km landscape to various predictors. Night was specified as baseline. β0 = intercept. β1 = area of agriculture within the landscape. β2 = density of non-avoided twigs within the landscape. ":" denotes an interaction. RMU = random effect for Regional Management Unit.

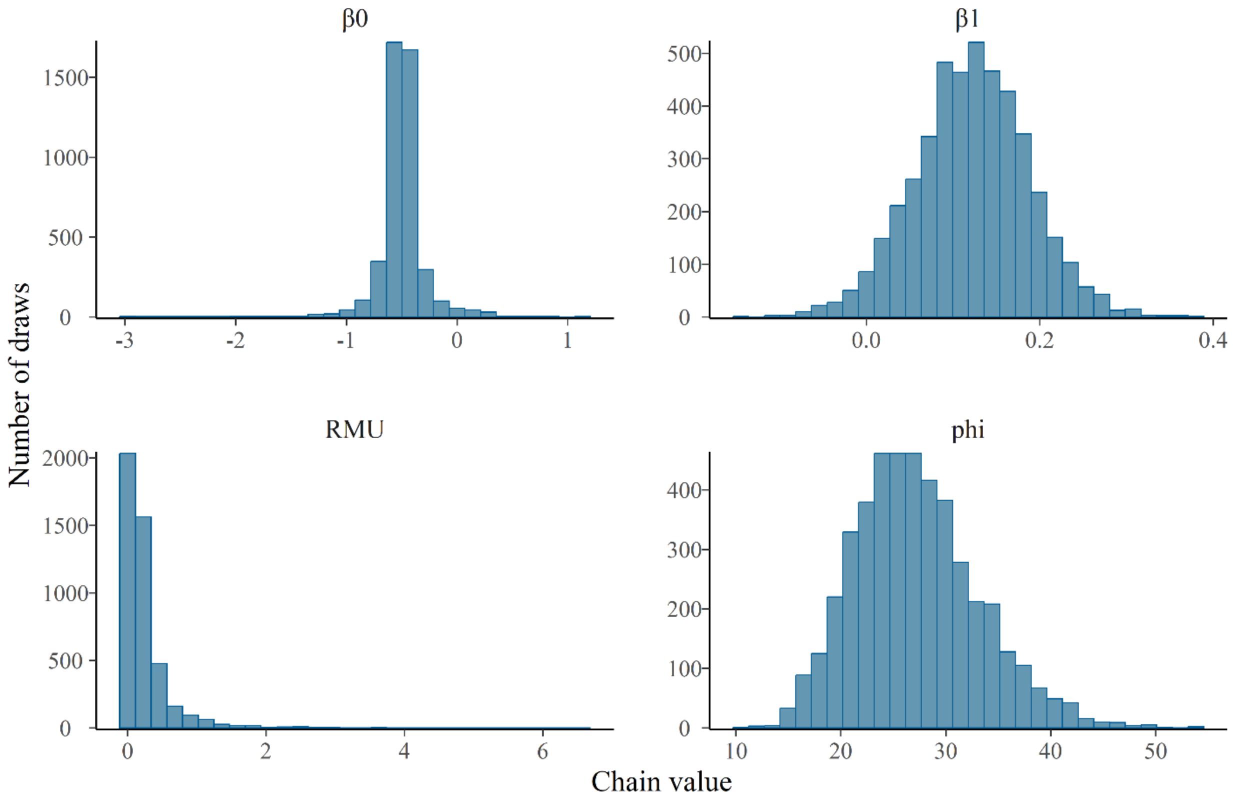

Supplementary Figure S3. Posterior distributions of a Bayesian beta regression model predicting activity levels of white-tailed deer within 3.2 x 3.2-km landscapes as a function of the number of buildings within the same landscapes. β0 = intercept. β1 = the number of buildings. RMU = random effect for Regional Management Unit.

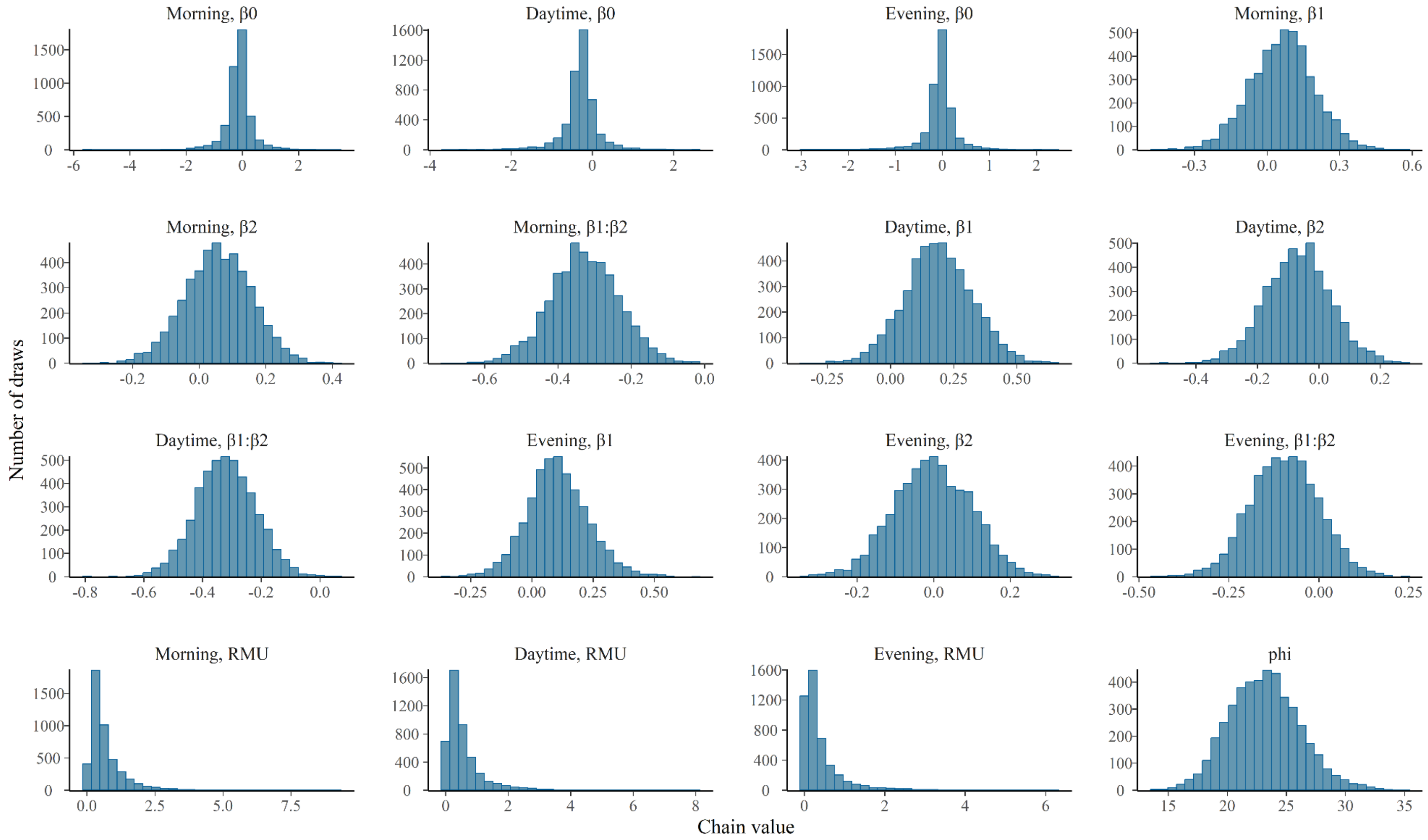

Supplementary Figure S4. Posterior distributions of a Bayesian beta regression model predicting activity levels of white-tailed deer exerted within morning, daytime, evening, and night within a 3.2 x 3.2-km landscape as a function of an interaction between the area of agriculture and density of non-avoided twigs within the landscape. β0 = intercept. β1 = area of agriculture within the landscape. β2 = density of non-avoided twigs within the landscape. ":" denotes an interaction. RMU = random effect for Regional Management Unit.

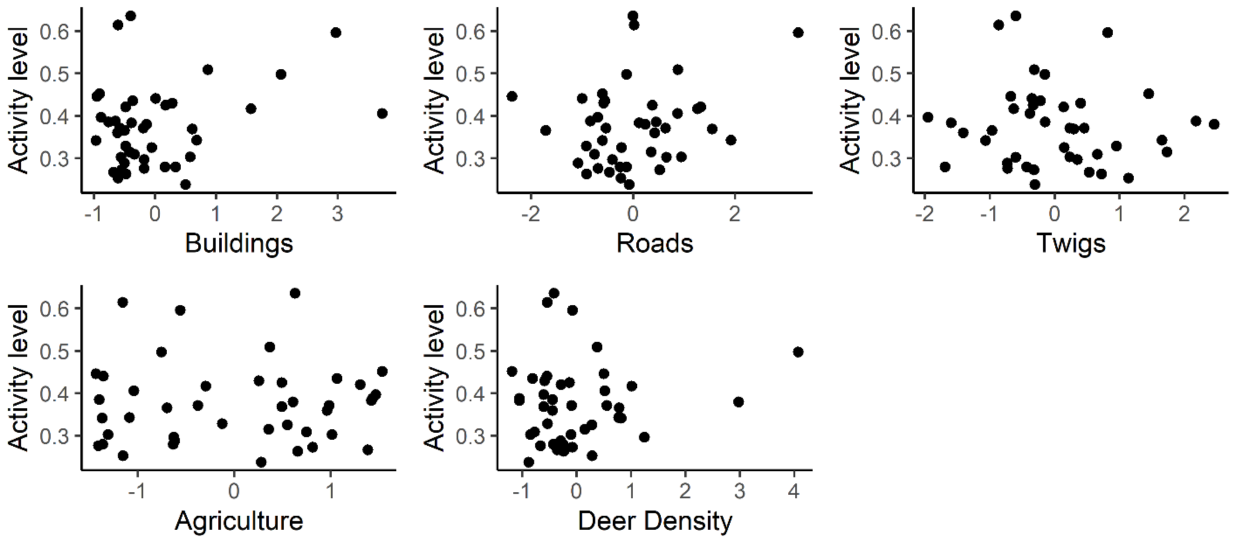

Supplementary Figure S5. Scatterplots of estimated activity levels in each replicate landscape in relation to the number of buildings, length of roads, density of twigs, area of land used for agriculture, and deer density in the same landscapes. All environmental covariates are standardized (i.e., subtracted the mean and divided by the standard deviation).

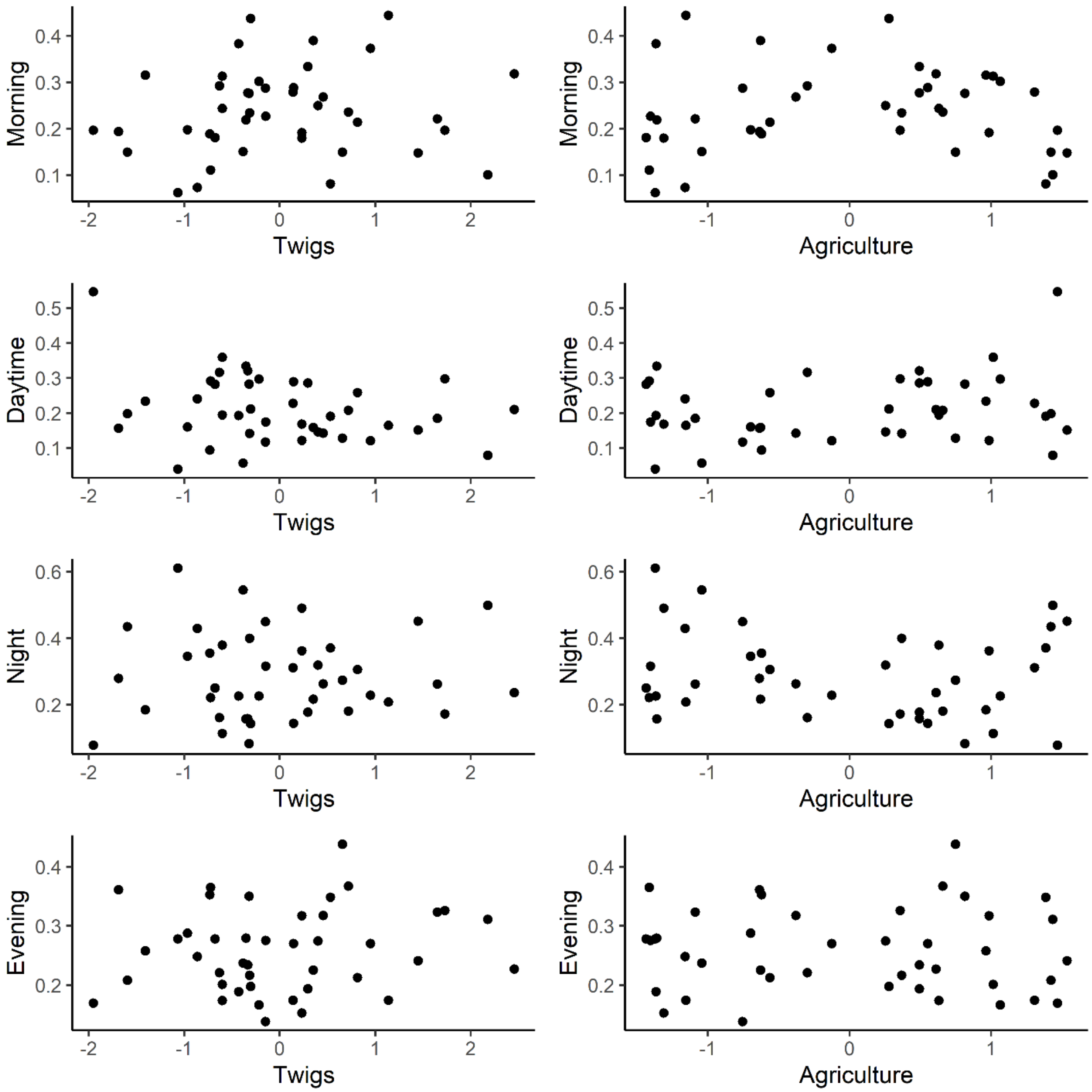

Supplementary Figure S6. Scatterplots of the estimated fractions of activity exerted during morning, daytime, night, and evening in each replicate landscape in relation to the density of twigs and area of land used for agriculture in the same landscapes. All environmental covariates are standardized (i.e., subtracted the mean and divided by the standard deviation).

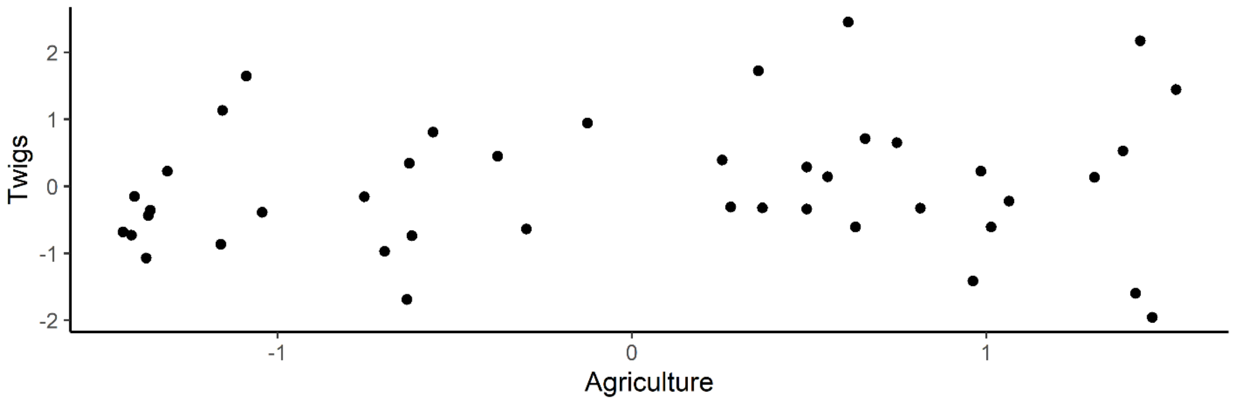

Supplementary Figure S7. Scatterplots of environmental predictors used within interactions when modelling activity levels and patterns of white-tailed deer. Interactions included the density of twigs interacting with the area of land used for agriculture. All environmental covariates are standardized (i.e., subtracted the mean and divided by the standard deviation).

Supplementary Table S1. List of different models considered when modelling white-tailed deer activity levels and patterns as a function of different environmental covariates. Buildings = the number of buildings in the landscape (i.e., 3.2 x 3.2-km area in which camera traps were deployed). Road length = the length of roads within the landscape. Twigs = the density of non-avoided woody twigs in the landscape. Ag = the amount of land used for agriculture in the landscape. Deer density = the average density of deer in the landscape. “*” denotes an interactive and additive effect.

Response	Model form
Level	~Buildings
	~Road length
	~Twigs
	~Ag
	~Twig * Ag
	~ Deer density
	~Twigs * Ag * Deer density
	~Twigs * Buildings * Deer density
Pattern	~Twigs
	~Ag
	~Twig * Ag
	~Twigs * Ag * Deer density
	~Twigs * Buildings * Deer density
